# Visualizing the Fate of Intra‐Articular Injected Mesenchymal Stem Cells In Vivo in the Second Near‐Infrared Window for the Effective Treatment of Supraspinatus Tendon Tears

**DOI:** 10.1002/advs.201901018

**Published:** 2019-08-01

**Authors:** Yimeng Yang, Jun Chen, Xiliang Shang, Zhujun Feng, Chen Chen, Jingyi Lu, Jiangyu Cai, Yuzhou Chen, Jian Zhang, Yuefeng Hao, Xing Yang, Yunxia Li, Shiyi Chen

**Affiliations:** ^1^ Department of Sports Medicine Huashan Hospital Fudan University Shanghai 200040 China; ^2^ Department of Anatomy and Physiology School of Medicine Shanghai Jiao Tong University Shanghai 200025 China; ^3^ Department of Sports Medicine Shanghai Sixth People's Hospital Affiliated to Shanghai Jiao Tong University Shanghai 200233 China; ^4^ Department of Orthopedics Affiliated Suzhou Hospital of Nanjing Medical University Suzhou Jiangsu 215500 China

**Keywords:** intra‐articular injection, mesenchymal stem cells therapy, PbS quantum dots, second near‐infrared window, supraspinatus tendon tear

## Abstract

Mesenchymal stem cells (MSCs) are capable of exerting strong therapeutic potential for the treatment of supraspinatus tendon tear. However, MSC therapy remains underutilized and perhaps underrated due to the limited evidence of dynamic visualization of cellular behavior in vivo. Here, second near‐infrared fluorescence imaging with biocompatible PbS quantum dots (QDs) provides a cellular migration map and information on the biodistribution and clearance processes of three densities of intra‐articularly injected, labeled MSCs to treat supraspinatus tendon tear in mice. Intra‐articular injection avoids entrapment of MSCs by filter organs and reduces the QD‐induced organ toxicity. Notably, the MSCs share a similar migration direction, but the moderate density group is somewhat more efficient, showing the longest residence time and highest cell retention rate around the footprint during the repair stage. Furthermore, quantitative kinetic investigation demonstrates that labeled MSCs are cleared by feces and urine. Histomorphometric analysis demonstrates that the moderate density group achieves maximum therapeutic effect and labeled MSCs do not induce any injury or inflammation to major organs, which suggests that administration of too many or few MSCs may decrease their effectiveness. Such an imaging approach provides spatiotemporal evidence for response to MSC therapy in vivo, facilitating the optimization of MSC therapy.

## 1 Introduction

Supraspinatus tendon (ST) tear is a widespread disorder characterized by functional deficits in the shoulder such as weakness, decreased range of motion, and debilitating pain.^1^, ^2^, ^3^ Mesenchymal stem cell (MSC) therapy provides a unique opportunity for redirecting the healing process away from scar formation and toward the regeneration of a fibrocartilaginous tendon‐bone insertion (enthesis).^4^, ^5^ Akin to the use of pharmacokinetics for drug development, successful implementation of cell therapy will require a better understanding of cell fate and safety issues after transplantation, which can be achieved through the use of molecular imaging.^6^, ^7^, ^8^, ^9^, ^10^ In vivo monitoring will enable investigators to potentially direct cell migration, proliferation, and differentiation to improve efficacy. However, strategies to monitor the response of the transplanted stem cells following ST tear in vivo are still in their infant stages due to the limitations of imaging techniques and probe labeling, leaving us with many unanswered questions for the safe and effective administration of stem cell therapy.

Ideal molecular imaging should be noninvasive, highly sensitive, and capable of playing a more extensive role by evaluating strategies at the target tissue.^6^, ^11^ Optical fluorescence imaging possesses the potential of high spatial and temporal resolution, which is desired for basic biological research with animal models and subsequent clinical translation.^12^, ^13^ However, traditional fluorescence imaging in vivo in the traditional visible and first near‐infrared window (NIR‐I, 650–950 nm) has been superficial in depth with poor resolution, limited by tissue scattering of photons, autofluorescence, and photobleaching.^12^ The poor imaging depth and low signal‐to‐background ratio have hindered fluorescence imaging in preclinical and clinical settings.^14^

The advancement of fluorescence imaging techniques based on the second near‐infrared window (NIR‐II, 1000–1700 nm) inspired us to track cell fate to address the aforementioned challenges facing stem cell therapy. As a recent development, NIR‐II imaging techniques have shown an ability to provide excellent spatial and temporal resolution (25 µm and 20 ms), deeper tissue penetration (up to 3 cm) and better real‐time monitoring ability due to minimized scattering and reduced background autofluorescence in comparison with the fluorescence imaging in visible and the NIR‐I window.^15^, ^16^, ^17^, ^18^, ^19^, ^20^, ^21^ Despite being identified only several years ago, many exogenous imaging contrast agents with NIR‐II absorption or emission have been designed and developed, such as single‐walled carbon nanotubes (SWCNTs),^15^, ^22^ metal chalcogenide quantum dots (QDs, e.g., Ag_2_S QDs, PbS QDs),^11^, ^23^, ^24^, ^25^, ^26^, ^27^, ^28^ organic nanoparticles,^17^, ^29^, ^30^, ^31^ rare‐earth‐doped nanoparticles,^32^, ^33^, ^34^, ^35^, ^36^ and wide bandgap semiconducting QDs.^37^, ^38^ For example, Ag_2_S QDs‐based NIR‐II imaging is currently feasible and provides long‐term visualization of cell migration and distribution in vivo following cell transplantation.^11^, ^39^, ^40^, ^41^

However, MSCs trapped in filter organs such as the lungs and liver were far in excess compared to MSCs homing to the area of injury after intravenous delivery.^41^ The existence of the “critical zone,” an area of hypovascularity near the insertion of the ST,^42^, ^43^, ^44^ further decreases the quantity of MSCs that are able to reach the footprint. It is hoped that residual MSCs retained in the footprint are able to provide sufficient therapeutic benefit. Unfortunately, the mechanical stimuli derived from blood flow can trigger MSC differentiation in the “wrong” direction (endothelial and smooth muscle cells) rather than chondrogenic differentiation.^45^ Therefore, efficacy of a stem cell therapy for treating ST tear is hampered by intravenous delivery because of the substantial defect in cell retention and potential of perturbing chondrogenic differentiation capability.

In contrast to intravenous delivery, intra‐articular injection is expected to contribute to an increased cell retention rate near the footprint while reducing the potential harmful effects. Intra‐articular injection could not only avoid the sense of mechanical stimuli and trap many transplanted stem cells by organs, but also would not need to overcome the blood‐joint barrier to migrate to the injured region.^41^, ^45^, ^46^, ^47^ Thus, intra‐articular injection could decrease the necessary number of transplanted stem cells and reduce potential risk to organs that is derived from both MSCs and QDs.

Here, our prepared PbS QDs, peak emission at ≈1300 nm,^28^ were conjugated to Tat peptide, which is effective vehicle for labeling QDs entering MSC and then applied to track MSCs. Longitudinal monitoring of injected cells in vivo provided spatiotemporal migration maps of three densities of MSCs, allowing for interpretation of variation in the density response to stem cell therapy. The initiation time of migration, residence time and cell retention rate around the footprint during the repair stage may play an important role in the ability to exert a therapeutic effect. Meanwhile, these findings indicated that labeled MSCs could be cleared by feces and urine without inducing noticeable injury or inflammation in major organs. This work provided spatiotemporal migration maps and detail on the distribution and clearance of intra‐articular injected MSCs, which contributes to the overall understanding of the fate of injected cells to optimize cellular therapy.

## 2 Results and Discussion

**Figure 1:**
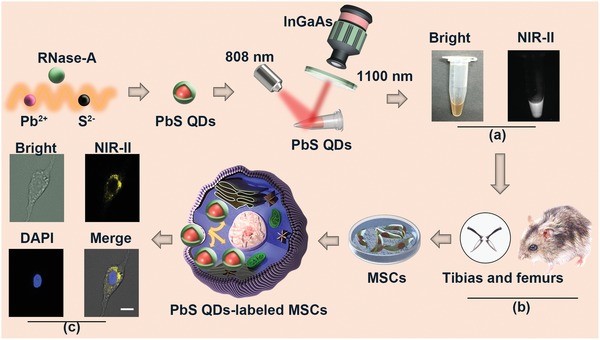
Schematic illustration of preparation and detection of NIR‐II signal in labeled MSCs. a) Bright field and NIR‐II fluorescence images of PbS QDs solution. b) Isolation of MSCs from mouse tibias and femurs. c) Bright field, NIR‐II fluorescence, diamidino‐phenyl‐indole (DAPI) fluorescence and merge photomicrographs of labeled MSCs. Scale bars represent 20 µm.

Our previously prepared PbS QDs with high quantum yield (17.3%) were first conjugated to Tat peptide. The water solution of PbS QDs successfully exhibited a strong NIR‐II fluorescence signal (**Figure** **1**a). Next, MSCs were isolated from mice tibias and femurs (Figure 1b), and investigation of the cellular uptake of the PbS QDs with bright field and NIR‐II fluorescence and 4′,6‐diamidino‐2‐phenylindole (DAPI) fluorescence were performed using a multichannel fluorescence microscope ranging from 400 to 1700 nm after incubation. As indicated in Figure 1c, photomicrographs demonstrated that PbS QDs were successfully internalized into MSCs to emit a strong NIR‐II signal. Moreover, dual fluorescence imaging demonstrated that the cellular uptake and accumulation of PbS QDs was mainly localized in the cytoplasm. These results demonstrated the promising potential of PbS QDs for NIR‐II fluorescence imaging with high signal‐to‐background ratio and the feasibility of applying PbS QDs to track MSCs.

**Figure 2:**
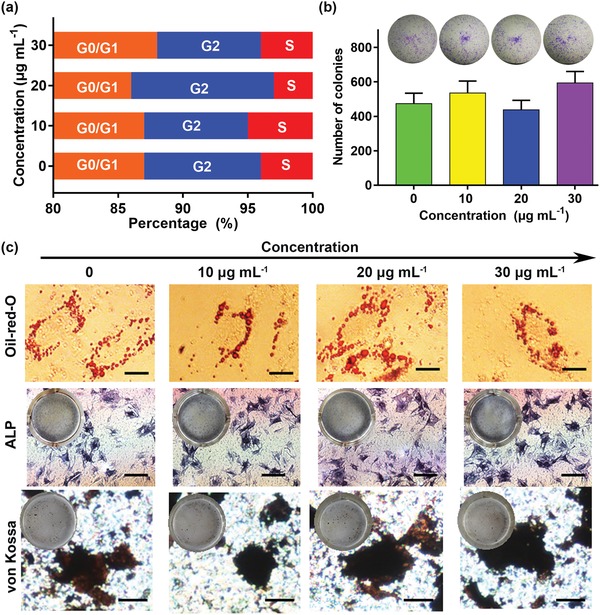
Interactions of different concentrations of PbS QDs with MSCs. a) Cell cycles detected by flow cytometry. b) Comparisons of colony‐formation efficiency in each group. c) Adipogenic and osteogenic differentiation assays for labeled MSCs, scale bars represent 20 µm (oil‐red O staining), 200 µm (ALP staining) and 50 µm (von Kossa staining).

MSCs exert their therapeutic effects for regeneration in part due to their multipotent capacity to direct differentiation toward a tendon or fibrocartilage phenotype.^48^, ^49^ The cell cycle has been found to influence cell fate transitions of pluripotent cells in perspectives other than proliferation.^50^, ^51^ The S and G2 phases attenuate pluripotent state dissolution because they possess an intrinsic propensity toward the pluripotent state that is independent of the G1 phase.^52^ Therefore, to assess the labeled MSCs multipotential differentiation, a cell cycle assay was carried out. No discernible changes in the cell cycle of MSCs labeled with different concentrations of PbS QDs were observed (*p* > 0.05) (**Figure** **2**a and Figure S1, Supporting Information). Furthermore, considering the colony‐forming assay has been long regarded as an accurate reflection of pluripotent stem cell activity, it is necessary to characterize the self‐renewal potential of the labeled MSCs. The colonies were homogeneous in size and morphology and negligible difference in the number of colonies (*p* > 0.05) was detected, potentially providing evidence that the labeled MSCs exhibited extensive self‐renewal capacity (Figure 2b). To further evaluate the potential impacts on differentiation capability of MSCs induced by PbS QDs, adipogenic and osteogenic differentiation assays were performed. Figure 2c shows that labeled MSCs were successfully differentiated into osteoblast and adipocyte lineages at a PbS QDs concentration of up to 30 µg mL^−1^. Likewise, MSCs treated with different concentrations of PbS QDs had similarly sized intracellular lipid‐rich vacuoles, similar alkaline phosphatase (ALP) activity and similarly sized calcium deposits in the extracellular matrix. These results illustrated that PbS QDs did not affect the multipotentiality of MSCs, which play a prominent role in tracking the labeled MSCs in vivo.

To characterize the proliferation of MSCs after incubation with PbS QDs, a live/dead staining assay and a cell counting kit‐8 (CCK‐8) assay was performed. A representative image demonstrated that almost all of the cells in each group were alive (Figure S2a, Supporting Information). There were no significant differences between the groups in MSC morphology or cell viabilities (*p* > 0.05), which were 94.6 ± 0.52% (0 µg mL^−1^ PbS QDs), 96.9 ± 0.89% (10 µg mL^−1^ PbS QDs), 97.9 ± 0.45% (20 µg mL^−1^ PbS QDs), and 97.2 ± 0.76% (30 µg mL^−1^ PbS QDs) (Figure S2b, Supporting Information). Similarly, no statistical variation was observed between 10 µg mL^−1^ group and other groups in the same time point (*p* > 0.05) (Figure S2c, Supporting Information). These results indicated that PbS QDs had negligible toxicity on MSC proliferation at concentrations from 10 to 30 µg mL^−1^.

**Figure 3:**
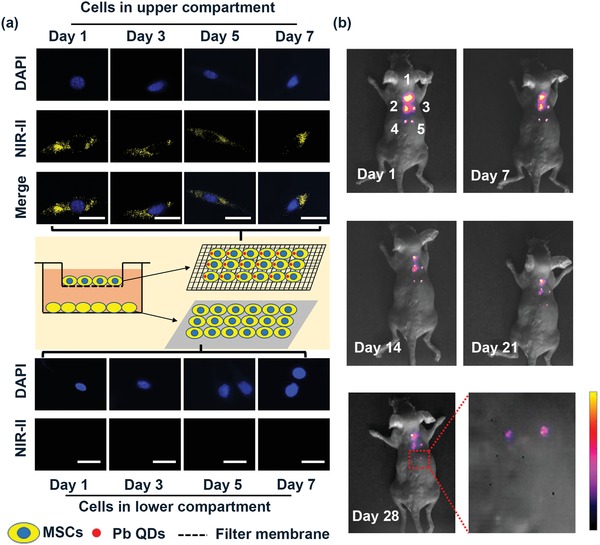
Detection the sensitivity and stability of labeled MSCs. a) Detection stability of labeled MSCs in vitro, scale bars represent 20 µm. b) Detection sensitivity and stability of different densities of labeled MSCs in vivo (1–2 × 10^5^ MSCs, 2–2 × 10^4^ MSCs, 3–5 × 10^3^ MSCs, 4–2 × 10^3^ MSCs, 5–1 × 10^3^ MSCs).

Leakage of intracellular fluorescent probes over time present a common potential drawback in MSC tracking and uptake of these nanoparticles by adjacent cells can occur and causes false positive signals.^53^, ^54^ Needless to say, investigation of PbS QDs leaking in vitro using a transwell culture system is required before tracking the fate of the transplanted stem cells in vivo. As shown in **Figure** **3**a, the NIR‐II signal of the labeled cells cultured in the upper compartment was clearly observed and steady within the tested time period, while unlabeled cells cultured in the lower compartment showed no NIR‐II signal within 7 days. Therefore, little to no leakage from labeled MSCs could alleviate the source of the interference in the NIR‐II signal when applying this strategy to track MSCs in vivo.

Although the imaging strategy worked well in vitro, it is necessary to further validate the sensitivity and stability of labeled MSCs in vivo before using PbS QDs to track transplanted MSCs in living animals. Therefore, multiple densities of labeled MSC populations (1 × 10^3^, 2 × 10^3^, 5 × 10^3^, 2 × 10^4^, and 2 × 10^5^ cells) were subcutaneously injected into the dorsum of a mice and NIR‐II fluorescence images were collected. Figure 3b shows that NIR‐II signal increased as a function of labeled MSC density, with an *R* ^2^ of 0.98 for this in vivo imaging analysis (Figure S3, Supporting Information). Interestingly, the finding showed that at least 1 × 10^3^ labeled MSCs could be readily detected using the in vivo imaging system, providing the possibility of visualizing the whole‐body migration and distribution of the transplanted cells with high sensitivity (Figure 3b). The detection limit is much lower than what was previously reported for CdSe@ZnS 655, emitting in the visible region (5 × 10^4^ cell), which highlights the sensitivity of PbS QDs contrast agents.^11^, ^55^, ^56^ The stability of NIR‐II fluorescence of PbS QDs was beneficial and the fluorescence signals in all densities of MSCs within 28 days postinjection were clearly detected, verifying that PbS QDs labeling was robust for long‐term in vivo tracking (Figure 3b and Figure S4, Supporting Information). Thus, the desirable sensitivity and stability of PbS QDs‐based NIR‐II imaging in vitro and in vivo shows that it is a promising candidate for in vivo visualization of the long‐term translocation and distribution of MSCs.

**Figure 4:**
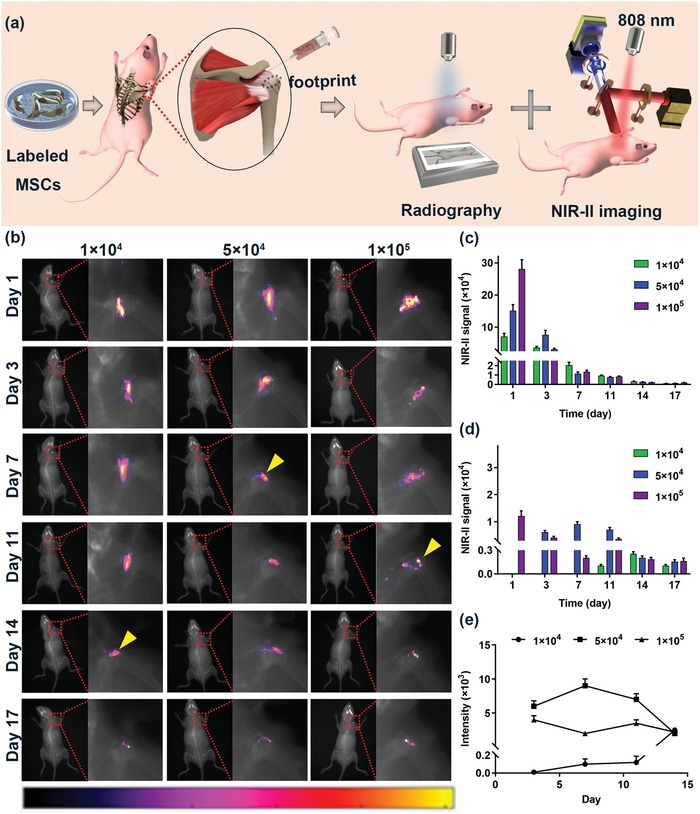
In vivo migration and distribution of intra‐articular injected MSCs. a) Schematic illustration of the radiography and NIR‐II imaging strategy. b) The time course of the migration and distribution of labeled MSCs; the yellow arrows indicated over 50% labeled MSCs started to retain near footprint. c) Quantitative measurement of the whole NIR‐II signal intensity in each group. d) Quantitative measurement of the NIR‐II signal intensity near the footprint. e) The time course of NIR‐II signal intensity near the footprint during enthesis repair stage.

Next, mice were subjected to unilateral detachment of the ST followed by intra‐articular injection of MSCs (Figure S5a–e, Supporting Information). The mice were assigned to 3 groups based on the density of the injected MSCs: 1) low density (1 × 10^4^ MSCs); 2) moderate density (5 × 10^4^ MSCs) and 3) high density (1 × 10^5^ MSCs). After surgery, micro CT analysis confirmed that the anterior and posterior tunnels were located in the footprint (native ST insertion area in the greater tuberosity, Figure S5f–j, Supporting Information). Both the radiography and NIR‐II images of the mice were collected synchronously for further visualization of whole‐body migration and distribution (**Figure** **4**a). The fluorescence intensity was found to exhibit linear behavior with cell density (*R* ^2^ = 0.98, Figure S6, Supporting Information).

As shown in Figure 4b, a strong NIR‐II signal was clearly observed in the mice shoulder, while filter organs such as the liver, lung, spleen, and kidney exhibited subtle NIR‐II signal intensity in vivo within 17 days, indicating that the joint capsule and the blood‐joint barrier reduced the tendency of MSCs to escape from the cavity. It is well known that QDs can cause DNA damage, antioxidant depletion and oxidative stress, which leads to cell death and tissue degradation; this toxicity is largely dose‐dependent.^57^, ^58^, ^59^ Compared to intravenous delivery, intra‐articular injection avoided exposure of filter organs to high doses of QDs and thus potentially reduced the QD‐induced cytotoxicity. Furthermore, most of the labeled MSCs were instantly confined in the articular cavity after injection and the whole NIR‐II signal gradually decreased in contrast to the steady increase around the footprint with time, suggesting the persistent accumulation of the injected MSCs near the footprint (Figure 4c,d). It is worth noting that the enthesis healing process starts with an inflammation stage, which causes the release of numerous chemokines to promote cell chemoattraction.^60^, ^61^, ^62^ Therefore, we postulated that chemotactic factors released in the inflammatory stage might guide the migration of MSCs.

Despite the fact that the MSCs in each group displayed similar migratory direction, the initiation time of migration and the residence time were considerably variable. At 3 days postinjection, labeled MSCs in the moderate density group began to migrate from the articular cavity to the footprint as the NIR‐II signal was first visible around the footprint (Figure 4b). In contrast, in the low density and high density groups, the initiation of migration occurred at 7 days postinjection. Meanwhile, more and more labeled MSCs in the moderate density group were enriched around the footprint and increased to 82% at 7 days postinjection. Since then, over 80% of MSCs have been retained near the footprint within the tested time period (Figure 4c,d). In contrast, no more than 11.1% of MSCs in the low density group and 43.8% of MSCs in the high density group were retained near the footprint up to 14 days postinjection. It is worth noting that the enthesis healing undergoes 3 stages: inflammation (0–7 days), repair (5–14 days) and remodeling (>14 days).^62^ During the repair stage, numerous growth factors are expressed, including transforming growth factor‐β (TGF‐β) and bone morphogenetic protein (BMP), which are essential for cell differentiation into a chondrocyte phenotype in the repair stage of enthesis healing.^63^, ^64^, ^65^, ^66^ Therefore, it is reasonable to postulate that the greater the number of MSCs and the longer residence time retained in the footprint during the repair process are, the more MSCs that differentiate and integrate into the repaired tissue. Quantitative analysis further revealed that the area under the curve (AUC) in the moderate density group was 2.4‐fold higher than that of the high density group and 16.4‐fold higher than that of the low density group (*P* < 0.05, Figure 4e) during the repair stage. These results showed that a PbS QD‐based NIR‐II imaging strategy worked well in tracking the fate of MSCs in vivo. The moderate density group was somewhat more efficient, being characterized by the longest residence time and highest cell retention rate around the footprint during the repair stage.

**Figure 5:**
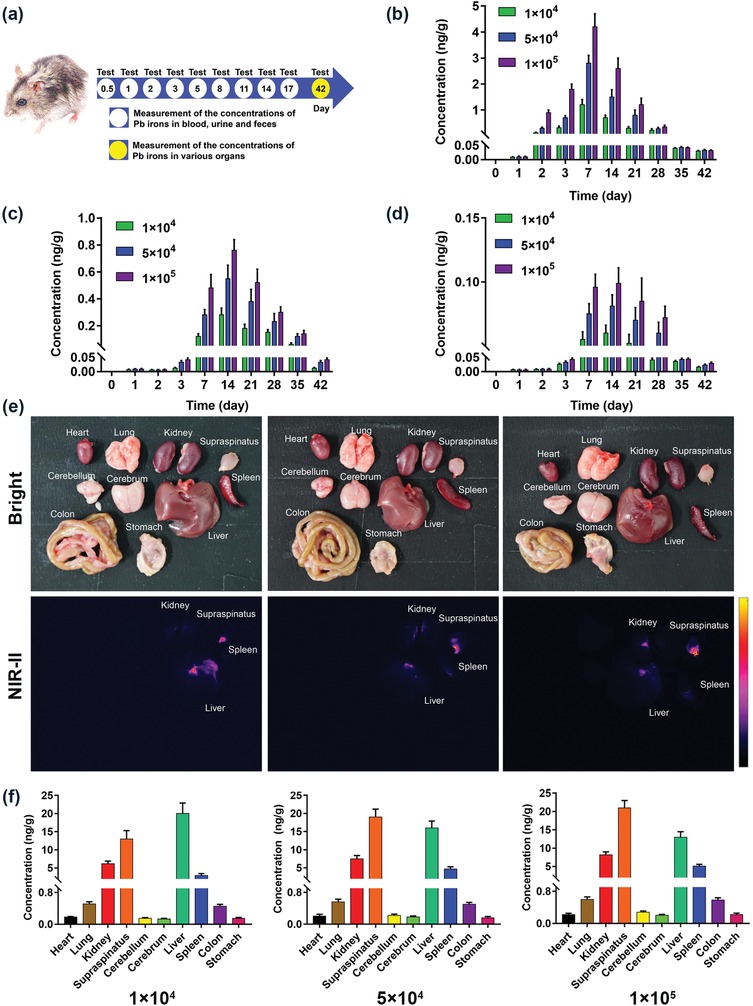
Quantitative analysis of the distribution and clearance processes of labeled MSCs in vivo. a) Schematic illustration of the study protocol of investigating the clearance of Pb ions. b) Determination of the concentration of Pb ions in whole blood. c) Determination of the concentration of Pb ions s in feces. d) Determination of the concentration of Pb ions in urine. e) Bright field and NIR‐II fluorescence images of various organs collected from the mice at 42 days after postinjection. f) Determination of the concentration of Pb ions in major organs.

Despite the fact that the imaging results enabled its practicability and potential in tracking the fate of MSCs and imaging‐guided cell therapeutics, quantitative analysis of labeled MSC distribution and clearance will undoubtedly assist in improving targeting efficiency of stem cell therapy. Therefore, the focus here was to quantitatively elucidate the in vivo kinetics and clearance of labeled MSCs by measuring the concentrations of Pb ions at various time points in whole blood, excretions and major organs (**Figure** **5**a). Figure 5b shows that there was no detectable Pb ions in whole blood until 2 days postinjection, and a significant increase was observed between 2 and 7 days postinjection. Subsequently, the concentration of Pb ions gradually decreased. Similarly, detectable Pb ions in excretions were observed at 3 days postinjection followed by an increase in the concentration of Pb ions from 3 to 14 days (Figure 5c,d). After that the concentration of Pb ions in excretions has gradually decreased. Meanwhile, we noticed that the concentration of Pb ions in feces was higher than that in urine within the tested time period, suggesting that feces was the principal pathway of clearance postinjection.

Further systemic investigations are required to understand the retention of labeled MSCs in the various organs after leaving the bloodstream. As shown in Figure 5e, most of the organs, especially the nervous system, showed subtle NIR‐II signal at 42 days postinjection, except for the liver, spleen, kidney, and supraspinatus muscle and tendon. The supraspinatus muscle and tendon exhibited clear NIR‐II signal in all three groups (Figure S7, Supporting Information), which confirmed that the injected cells have migrated and retained near the footprint.

**Figure 6:**
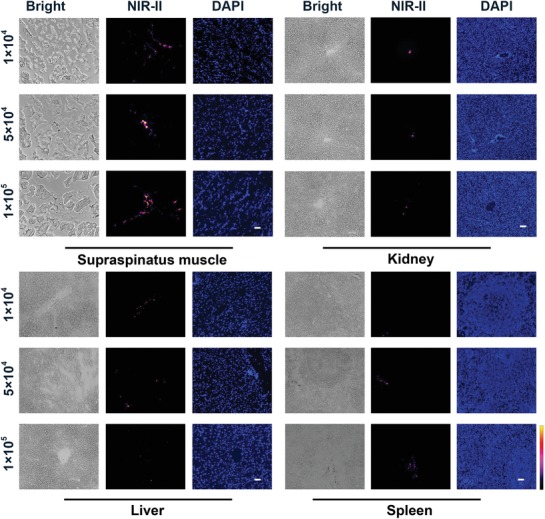
Representative bright field, NIR‐II fluorescence and DAPI fluorescence photomicrographs images of supraspinatus muscle, kidney, liver and spleen at 42 days after postinjection, scale bars represent 100 µm.

Additionally, the potential toxicity to organs was studied by measuring the content of Pb ions from various organ samples. As shown in Figure 5f, up to 21 ng g^−1^ of Pb was observed in the liver, spleen, kidney and supraspinatus muscle, which aligned with the NIR‐II fluorescence images. Furthermore, little to no retention of Pb ions was observed in other major organs, suggesting reduced QD‐induced organs toxicity. Moreover, a frozen section image was taken to further confirm the presence of labeled MSCs in the liver, spleen, kidney and supraspinatus muscle while NIR‐II signal was not observed in other organs (**Figure** **6** and Figure S8, Supporting Information).

**Figure 7:**
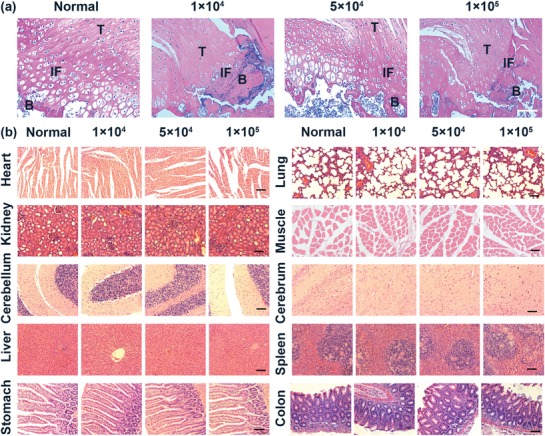
Therapeutic potential and toxicity of labeled MSCs. a) Representative photomicrographs of H&E staining on tendon‐to‐bone insertions from normal mice and MSCs treated mice, scale bars represent 100 µm, (T, tendon; B, bone; IF, interface). b) Representative photomicrographs of H&E staining on the major organs, scale bars represent 100 µm.

The therapeutic potential and the toxicity of the three densities of labeled MSCs were further evaluated by hematoxylin and eosin (H&E) staining. The representative images demonstrated the tendon‐bone insertion connectivity and increased cell densities in the moderate density group, whereas a gap at the insertion was observed in the low density and high density groups (**Figure** **7**a). Likewise, histological scoring evaluations proved to be the most efficacious in the moderate density group compared to the other two groups (*P* < 0.05, Figure S9, Supporting Information), suggesting the superiority of longer residence time and higher cell retention rate around the footprint. In addition, no noticeable injury or inflammation was observed in the H&E stained organ tissues (Figure 7b), which further proved the negligible toxicity of labeled MSCs. These findings demonstrated that the administration of too many or few MSCs may decrease their effectiveness, potentially suggesting that the density response to stem cell therapy is related to their diverse migration patterns and further indicating the existence of an optimal density.

## 3 Conclusions

In conclusion, PbS QDs are an excellent NIR‐II fluorescence contrast and are capable of labeling MSCs without affecting their proliferation, self‐renewal or differentiation capabilities. Intra‐articular injected MSCs were instantly blocked in the articular cavity after injection and gradually migrated to the footprint. Meanwhile, joint capsule and the blood‐joint barrier allowed for slow transport of MSCs to liver, spleen, and kidney via the blood, and then MSCs were cleared by feces and urine. Despite the fact that the MSCs in each of the three groups presented a similar migratory direction, the initiation time of migration, residence time and cell retention rate around the footprint were considerably variable. Taking into consideration the pathophysiological process of enthesis healing, the moderate density group was advantageous due to its longest residence time and highest cell retention rate around the footprint during the repair stage, which was confirmed by histological analysis. Histological analysis demonstrated labeled MSCs induced neither injury nor inflammation to major organs and the moderate density group proved to be the most efficacious, suggesting the existence of an optimal density of MSC for use in stem cell therapy. This work provides a spatiotemporal migration map of MSCs and information on their distribution and clearance following intra‐articular injection, which will lead to more focused studies for optimization and clinical translation of cellular therapy.

## 4 Experimental Section

*Cell Labeling by Tat‐PbS QDs*: Highly fluorescent PbS QDs were prepared by coating PbS QDs with Ribonuclease‐A as described in the previous study.^28^ PbS QDs were conjugated to Tat peptide through covalent bonding by using the cross linking reagent Sulfo‐SMCC (Sigma).^11^, ^67^ MSCs were isolated from tibias and femurs of mice and were incubated with α‐MEM medium containing Tat‐PbS QDs (10 µg mL^−1^) for 12 h.^11^, ^68^ After incubation, MSCs were washed twice with phosphate buffered saline (PBS) to remove unbound free Tat‐PbS QD nanoparticles. For dual labeling of MSCs with Tat‐PbS QDs and DAPI, Tat‐PbS QD labeled MSCs were fixed with 4% paraformaldehyde, and nuclei were stained with DAPI (4 mg mL^−1^, Sigma) for 5 min. The Tat‐PbS QD labeled MSCs were incubated in α‐MEM medium containing 10% fetal bovine serum (FBS) (HyClone, UT, USA) for following experiments. The NIR‐II fluorescence images of Tat‐PbS QDs were collected using the NIR in vivo imaging system (Suzhou NIR Optics Technology Co., Ltd., China) equipped with an InGaAs/ shortwave infrared (SWIR) CCD camera (Photonic Science, UK), an 880 nm longpass filter and an 1100 nm longpass filter (Daheng Optics and Fine Mechanics Co., Ltd, China).^11^ An 808 nm diode laser (Starway Laser Inc., China) was used as the excitation source. The laser power density was 123.8 mW cm^−2^ (beam size 10^6^ cm^2^) during imaging, with an exposure time of 50 ms. The dual‐labeled MSCs were imaged by a multichannel NIR‐II fluorescence microscope (Suzhou NIR‐Optics Technology Co., Ltd., China).

*Cell Cycle Analysis of Tat‐PbS QDs Labeled MSCs*: The MSCs labeled with different concentrations of Tat‐PbS QDs (0, 10, 20, and 30 µg mL^−1^) were harvested at passage 3. At least 5 × 10^6^ Tat‐PbS QD labeled MSCs in each group were fixed with ice‐cold ethanol (95%) overnight at 4 °C, and then the fixed cells were washed twice with PBS. Cells were incubated with propidium iodide (40 mg mL^−1^, PI) staining solution and RNase A (10 mg mL^−1^) at 4 °C for 30 min. The PI‐elicited fluorescence of individual cells was measured using flow cytometry (Beckman Coulter).

*Colony‐Forming Assay of Tat‐PbS QDs Labeled MSCs*: Tat‐PbS QDs (0, 10, 20, and 30 µg mL^−1^) labeled MSCs were diluted in α‐MEM medium containing 10% FBS and were plated at ≈10 cells per cm^2^ in 6 well plates. After incubation for 14 days, the cells were washed with PBS and stained with 0.5% crystal violet (Sigma) for 15 min at room temperature. Cells were washed with PBS twice and colonies (>50 cells) were counted under the microscope (Olympus, Japan).

*Osteogenic and Adipogenic Differentiation of Tat‐PbS QDs Labeled MSCs*: The differentiation medium was prepared as described in the previous study.^66^ For osteogenic differentiation, MSCs labeled with different concentration of Tat‐PbS QDs (0, 10, 20, and 30 µg mL^−1^) were cultured in a α‐MEM and 10% FBS medium containing dexamethasone (10^−7^ m), β‐glycerol phosphate (10 × 10^−3^ m) and ascorbate‐2‐phosphate (50 × 10^−6^ m). Osteogenic cells were determined by ALP staining and von Kossa staining at 14 and 28 days after induction, respectively. For adipogenic differentiation, cells were incubated for 2 weeks in α‐MEM and 10% FBS medium containing dexamethasone (10^−6^ m), isobutylmethylxanthine (0.5 × 10^−6^ m) and insulin (10 ng mL^−1^). Adipogenic cells were determined by Oil Red O staining at 14 days after induction. Both adipogenic and osteogenic cells were imaged under a microscope.

*Proliferation of Tat‐PbS QDs Labeled MSCs*: MSCs labeled with different concentrations of Tat‐PbS QDs (0, 10, 20, and 30 µg mL^−1^) were cultured in 24 well plates (2 × 10^3^). CCK‐8 solution (Dojindo, Japan) at a ratio of 100 µL/1 µL was added to the plates at days 1, 3, 7, 9, 11, 15, and 17 days after labeling. The plates were incubated at 37 °C for 1 h and absorbance was then measured at a wavelength of 450 nm using a microplate reader. Five wells from each group at each time point were assayed. The live/dead staining assay was performed 7 days after labeling. In brief, the MSCs were washed three times with PBS and incubated in calcein AM (2 × 10^−6^ m, staining live cells) and PI (8 × 10^−6^ m, staining dead cells) in PBS for 30 min at 37 °C and washed again with PBS. A fluorescence microscope (Olympus, Japan) was used to image the samples. The images were further processed by ImageJ software to count viable cells.

*Detection Sensitivity and Stability of Tat‐PbS QDs Labeled MSCs*: The leakage of Tat‐PbS QDs through exocytosis was determined using a transwell culture system.^69^ 1 × 10^5^ MSCs labeled with Tat‐PbS QDs (10 µg mL^−1^) were cultured in the upper compartment of the transwell culture system, while 1 × 10^5^ unlabeled MSCs were grown in the lower compartment. The upper and lower compartments were separated by a porous membrane (8 µm, Corning) that allowed the passing of free nanoparticles but not cells. MSCs were harvested at day 1, 3, 5, and 7 days after cultivation and stained with DAPI. MSCs were imaged using a multichannel NIR‐II fluorescence microscope.

Six‐week‐old female Balb/c mice were purchased from Shanghai Jiesijie Laboratory Animal Corporation. Animal studies were performed under the guidelines approved by Fudan University. The Tat‐PbS QDs (10 µg mL^−1^) labeled MSCs (1 × 10^3^, 2 × 10^3^, 5 × 10^3^, 2 × 10^4^, and 2 × 10^5^ cells) in PBS (20 µL) were subcutaneously injected into the dorsum of mice. NIR‐II fluorescence images of the mice were collected at days 1, 7, 14, 21, and 28 after injection. All the images were collected using an NIR in vivo imaging system as described above with an exposure time of 100 ms. The calibrated NIR‐II fluorescent intensity of labeled MSCs was analyzed by ImageJ software.

*In Vivo Tracking of Intra‐Articular Injected MSCs in Mice with Supraspinatus Tendon Tear*: Mice were anesthetized by breathing isoflurane and the ST was detached from the greater tuberosity as previously described.^70^ After fixing the ST to the footprint, three densities of MSCs (1 × 10^4^, 5 × 10^4^, and 1 × 10^5^ MSCs) labeled with Tat‐PbS QDs (10 µg mL^−1^) were intra‐articularly injected into the shoulder joint cavity. The humerus was scanned at a spatial resolution of 18 mm (1 mm aluminum filter, 65 kV, 378 mA) using a Skyscan 1176 micro‐CT imaging system (Bruker, Kontich, Belgium).

For in vivo imaging studies, both NIR‐II fluorescence images and radiography images were collected at 1, 3, 7, 11, 14, and 17 days after injection. All the NIR‐II fluorescence images were collected using an NIR in vivo imaging system as described above with an exposure time of 100 ms. For radiography, an optical and X‐ray small animal imaging system (In Vivo Xtreme, Bruker) was used with the following acquisition parameters: a field of view of 18 cm, f/2.0 and high speed mode. The mice were kept alive until 42 days after injection. The calibrated NIR fluorescent intensity of Tat‐PbS QD labeled MSCs in the whole body and footprint was analyzed using ImageJ software.

Whole blood (0.2 mL) was collected from the postorbital venous plexus of each mouse at predetermined times (1, 2, 3, 7, 14, 21, 28, 35, and 42 days after injection). Metabolism cages were used to collect urine and feces at the same predetermined times. The blood samples were centrifuged and supernatant (plasma) was subsequently analyzed for Pb ion concentration by inductively coupled plasma atomic emission spectroscopy (ICP‐AES) which was correlated to the PbS QD‐labeled MSCs and served as the basis in determining their toxicity. To prepare of the feces samples, a 3× excess weight of double‐distilled water (DD‐H_2_O) was added to facilitate softening, soaking for two days.^71^ Urine samples were diluted with an equal volume of DD‐H_2_O.^71^ The plasma and excretion samples were dissolved in 5 mL digest solution (HNO_3_:HClO_4_ = 4:1) and heated to 220 °C for 1 h. The reaction was stopped when it became clear and the solution was then cooled down to room temperature.^72^ Each of the resulting solutions was then diluted to a final volume of 10 mL with DD‐H_2_O, and subsequently analyzed by ICP‐AES.

Mice were sacrificed carefully 42 days after injection. The organs, including the heart, lung, kidneys, supraspinatus muscle and tendon, cerebellum, cerebrum, liver, spleen, colon and stomach were collected and imaged using the NIR in vivo imaging system. The calibrated NIR fluorescence intensities of organ samples were analyzed by ImageJ software. The organ samples were divided it into two parts. A portion of the organ samples were prepared in a similar fashion as described above for ICP‐AES measurements. All the ICP‐AES measurements were repeated three times. The rest organ samples were stained with H&E and DAPI. For fluorescence microscopy analysis, samples were imaged with a multichannel NIR‐II fluorescence microscope. Specimens from the footprint were decalcified in 10% ethylenediaminetetraacetic acid for 2 weeks and dehydrated in a graded series of ethanol. After that samples were embedded in paraffin and cut into 5 µm sections and then stained with H&E. Images were obtained by using an Olympus microscope. A modified histologic scoring system was examined for tendon‐to‐bone interface characteristics including cell morphology, cellularity and vascularity as described in previous studies.^73^, ^74^ Three sequential sections were assessed to provide an average score per specimen.

*Statistical Analysis*: Quantitative data are expressed as the mean ± standard error of at least three determinations. Nonparametric data were analyzed with the Kruskal–Wallis test. Parametric data were compared by analysis of variance and the Tukey post hoc test. All statistical analyses were performed with SPSS 23.0 software. A *p* value <0.05 was considered to be statistically significant.

## Conflict of Interest

The authors declare no conflict of interest.

## Supporting information

Supplementary
