# Supplementary material for: Visualizing the Fate of Intra‐Articular Injected Mesenchymal Stem Cells In Vivo in the Second Near‐Infrared Window for the Effective Treatment of Supraspinatus Tendon Tears
Source: Adv Sci (Weinh). 2019 Aug 1;6(19):1901018. doi: 10.1002/advs.201901018 (PMC6774022; doi:10.1002/advs.201901018)
Supplement: Supplementary file 1 — Supplementary [file ADVS-6-1901018-s001.pdf]

Copyright WILEY-VCH Verlag GmbH & Co. KGaA, 69469 Weinheim, Germany, 2019.

ADVANCED SCIENCE Open Access

Supporting Information

for *Adv. Sci.,* DOI: 10.1002/advs.201901018

Visualizing the Fate of Intra-Articular Injected Mesenchymal Stem Cells In Vivo in the Second Near-Infrared Window for the Effective Treatment of Supraspinatus Tendon Tears

*Yimeng Yang, Jun Chen, Xiliang Shang, Zhujun Feng, Chen Chen, Jingyi Lu, Jiangyu Cai, Yuzhou Chen, Jian Zhang, Yuefeng Hao, Xing Yang, Yunxia Li, and Shiyi Chen**

Supporting Information

Visualizing the Fate of Intra-Articular Injected Mesenchymal Stem Cells In Vivo in the Second Near-Infrared Window for the Effective Treatment of Supraspinatus Tendon Tears

Yimeng Yang, Xiliang Shang, Zhujun Feng, Chen Chen, Jingyi Lu, Jiangyu Cai, Yuzhou Chen, Jian Zhang, Yunxia Li, Yuefeng Hao, Xing Yang, Jun Chen, and Shiyi Chen**

Dr. Y. Yang, X. Shang, J, Lu, J. Cai, Y. Chen, J. Zhang, Y. Li, J. Chen, Prof. S. Chen

Department of Sports Medicine, Huashan Hospital, Fudan University, Shanghai, 200040, China.

E-mail: cshiyi@163.com, biochenjun@fudan.edu.cn.

Z. Feng

Department of Anatomy and Physiology, School of Medicine, Shanghai Jiao Tong University, Shanghai, 200025, China.

C. Chen

Department of Sports Medicine, Shanghai Sixth People's Hospital Affiliated to Shanghai Jiao Tong University, Shanghai, 200233, China.

Y. Hao, X. Yang

Department of orthopedics, Affiliated Suzhou Hospital of Nanjing Medical University, Suzhou 215500, Jiangsu, China.

Keywords: mesenchymal stem cells therapy, intra-articular injection, supraspinatus tendon tear, PbS quantum dots, second near-infrared window

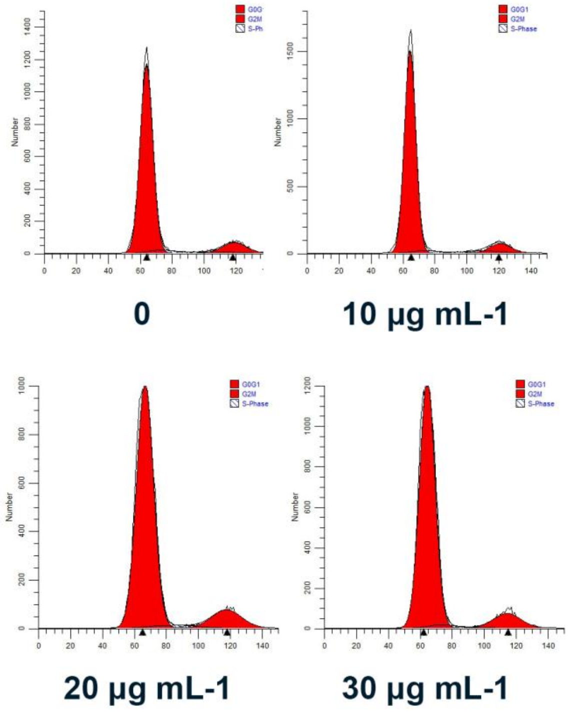

Figure S1. The effects of different concentrations of PbS QDs on cell cycle distribution.

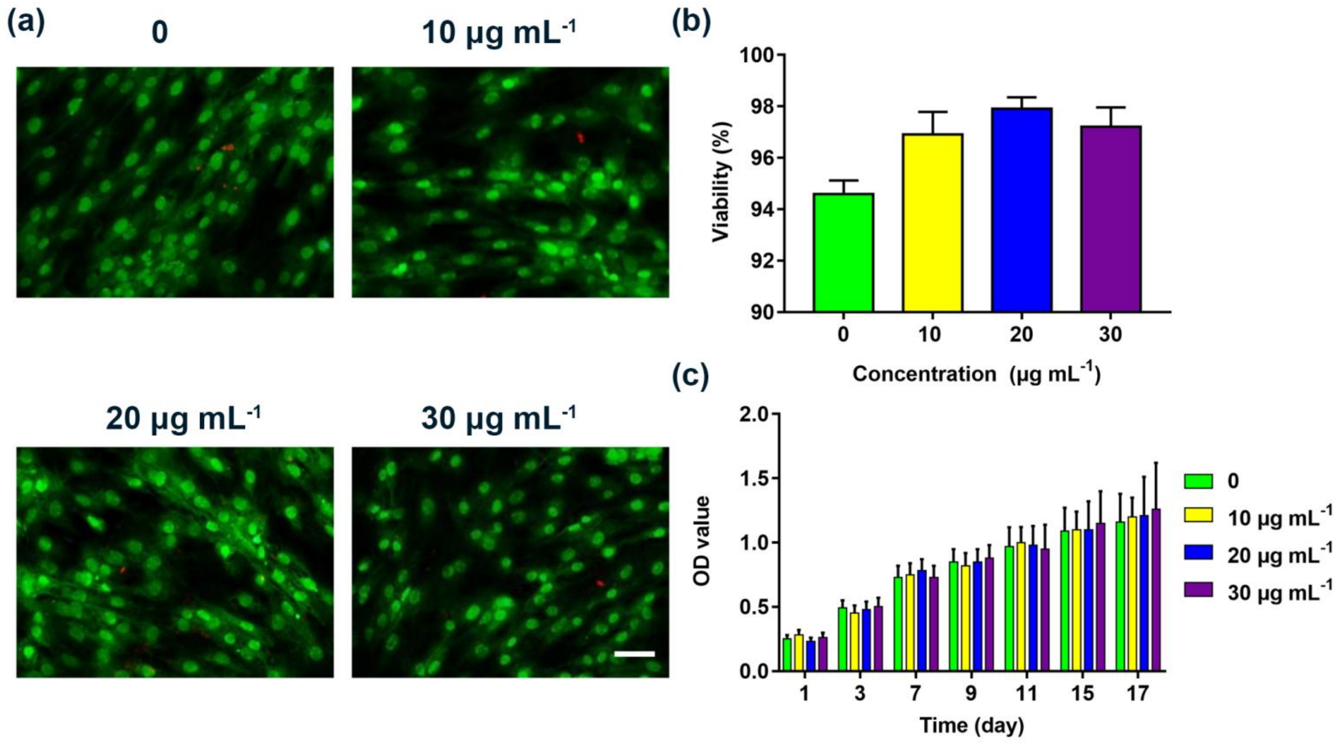

Figure S2. The effects of different concentrations of PbS QDs on cell proliferation and cell viability. (a). Live/dead cell staining for labeled MSCs (green fluorescence-live cells, red fluorescence-dead cells). Scale bars represent 50 μm. (b). Quantification analysis of cell viability of labeled MSCs. (c) Cell proliferation of labeled MSCs determined by CCK-8.

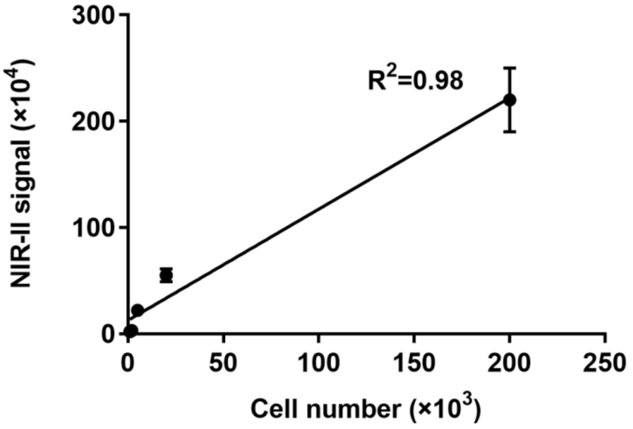

Figure S3. The linear relationship between cell density and NIR-II signal intensity after injecting labeled MSCs into the dorsum of mice.

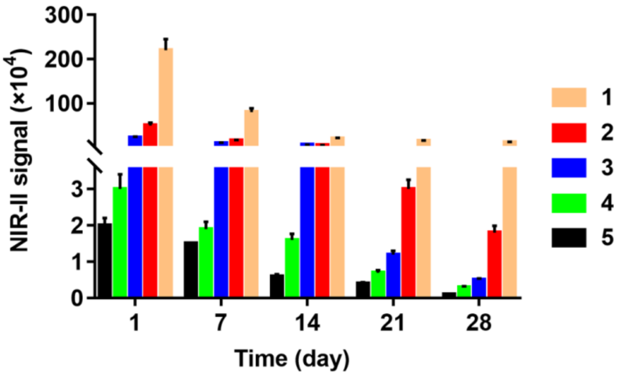

Figure S4. Quantification analysis of NIR-II signal intensity of labeled MSCs within 28 days after postinjection (1-2×10^5 MSCs, 2-2×10^4 MSCs, 3-5×10^3 MSCs, 4-2×10^3 MSCs, 5-1×10^3 MSCs).

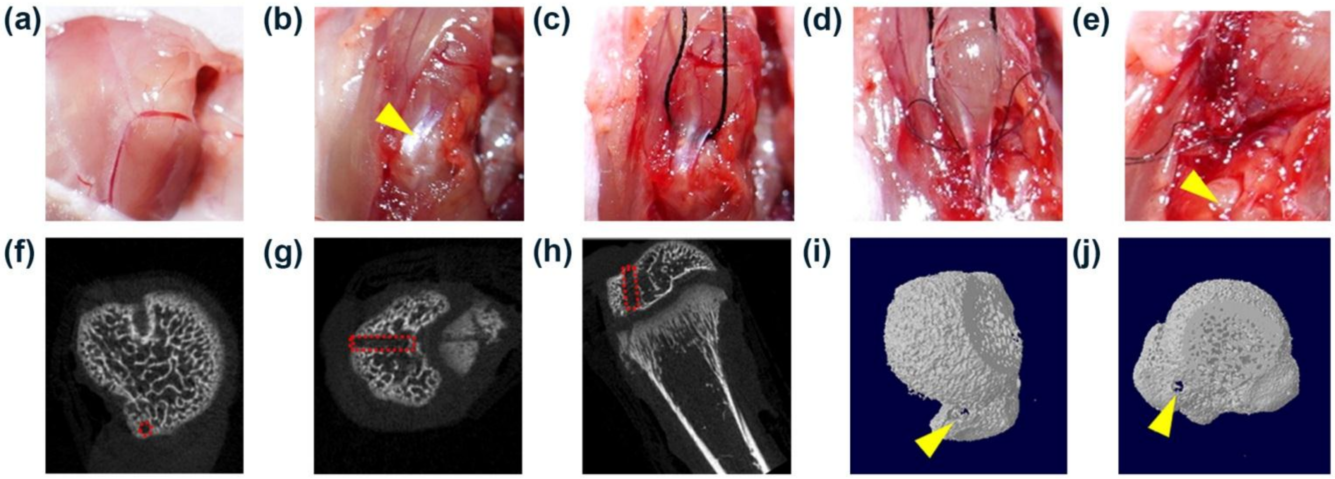

Figure S5. The surgical procedures of unilateral detachment of the ST and micro CT analysis for the position of tunnels. (a) The deltoid muscle was identified with the overlying vasculature identifying the borders of the muscle. (b) The deltoid muscle was minimally dissected to expose the ST; the yellow arrows indicated the ST. (c) A 5-0 Prolene suture was used to develop space under the supraspinatus. (d) A double-needled 8/0 Prolene was used to fashion a modified Kessler suture starting at the midsubstance of the tendon with one needle and completing the configuration with the other needle. (e) The tendon was then detached from the footprint; the yellow arrows indicated the footprint. (f) The position of the tunnel was confirmed in cross-section (f), sagittal planes (g) and coronal planes (h) by micro CT; the red circles indicated the tunnel. (i). 3D reconstruction of the humeral confirmed that the position of anterior (j) and posterior tunnel (k) were localized in the footprint; the yellow arrows indicated the tunnel.

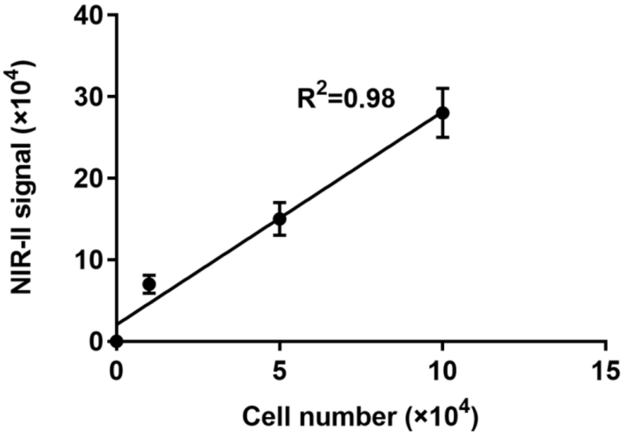

Figure S6. The linear relationship between cell density and whole NIR-II signal intensity after intra-articular injection.

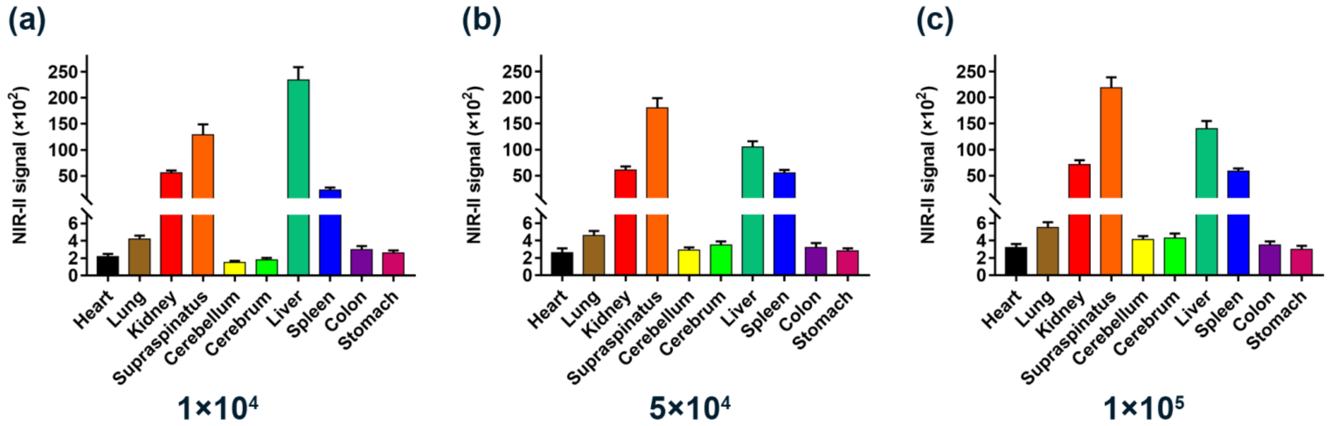

Figure S7. Quantitative analysis NIR-II signal intensity of the low density (a), moderate density (b) and high density groups (c) at 42 days after post-injection.

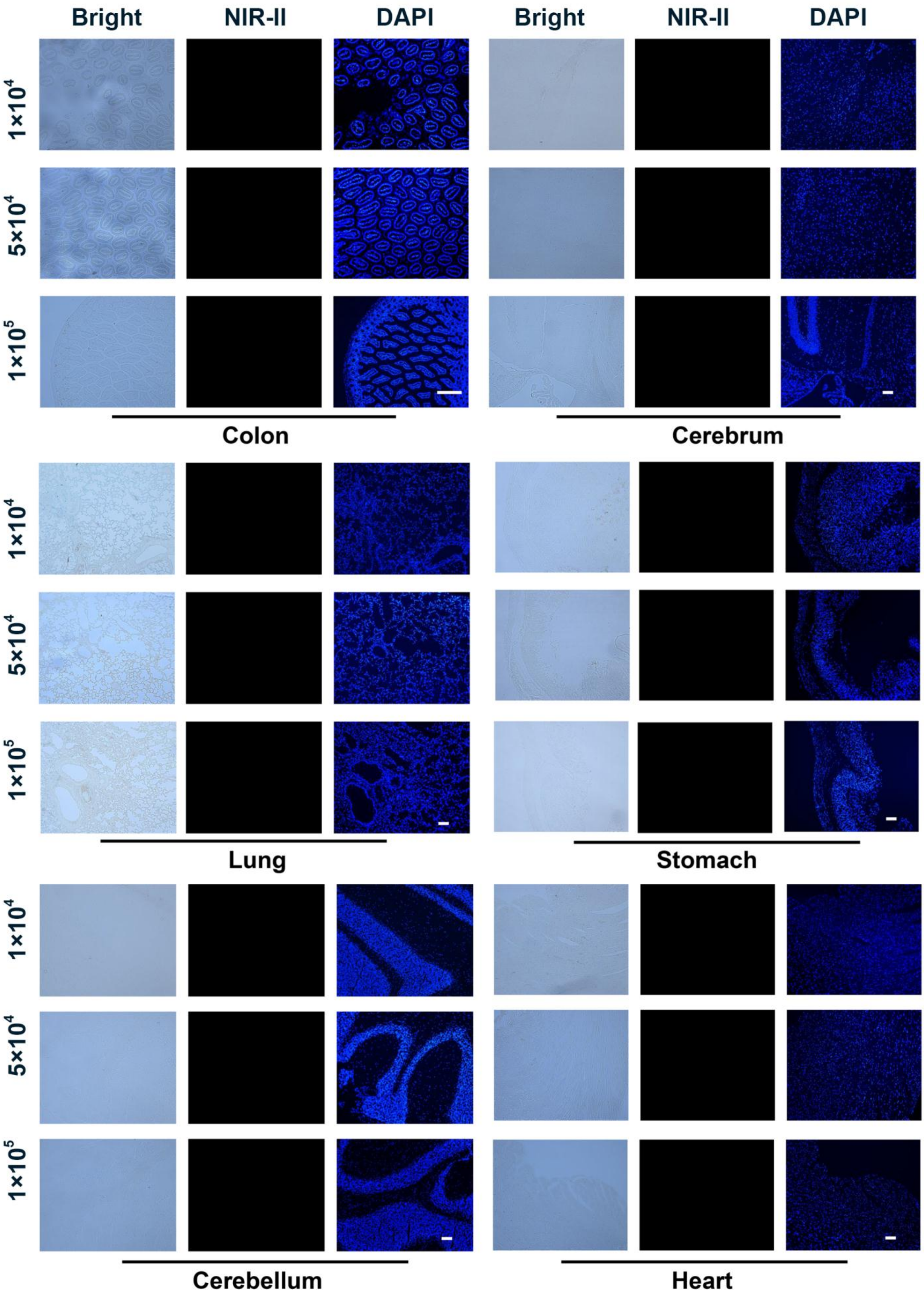

Figure S8. Representative bright field, NIR-II fluorescence and DAPI fluorescence images of various organs collected from each mice. Scale bars represent 100 μm

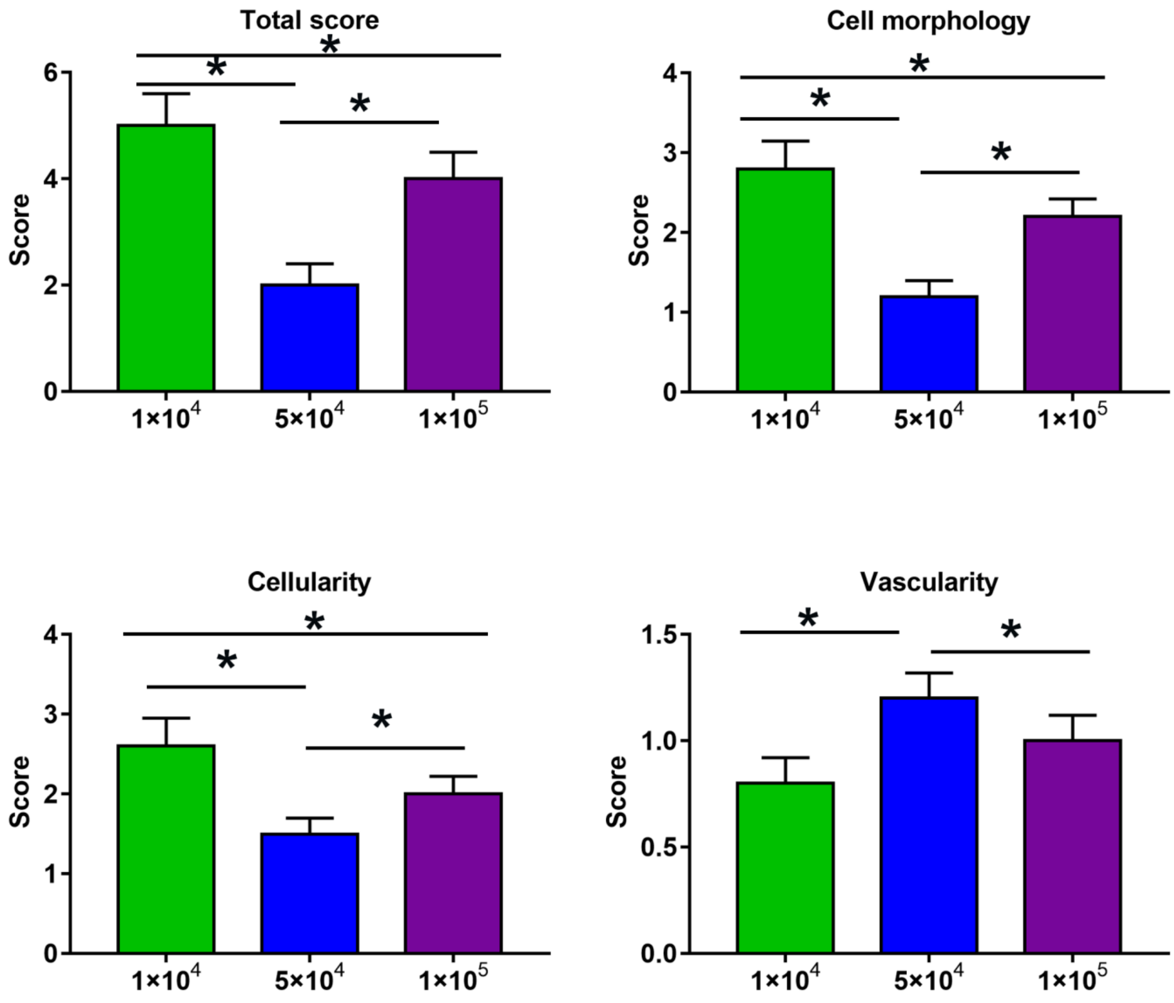

Figure S9. Histological examination scores of tendon-to-bone insertions in each group. *$P < 0.05$.
